# Case report: Highly response to low-dose brachytherapy in recurrent retroperitoneal leiomyosarcoma with FANCD2 frameshift mutation: a unique case study

**DOI:** 10.3389/fonc.2024.1339955

**Published:** 2024-04-03

**Authors:** Xiao li Liu, Jinxin Zhao, Xue min Di, Guohui Cao, Hongtao Zhang, Juan Wang

**Affiliations:** Department of Oncology, Hebei General Hospital, Shijiazhuang, China

**Keywords:** retroperitoneal leiomyosarcoma, FANCD2, brachytherapy, radioactive iodine-125, low dose

## Abstract

We report a case of recurrent retroperitoneal leiomyosarcoma in a male who achieved a rapid and robust but transient clinical response to low-dose iodine-125 brachytherapy. A *FANCD2* frameshift mutation was detected by gene sequencing in the cancerous tissue.

## Introduction

Retroperitoneal sarcoma (RPS) is a rare disease, and surgery is the mainstay of its treatment (1). Retroperitoneal leiomyosarcoma (RLMS) is a type of RPS. Recurrence after RPS resection, which occurs in more than half of patients, is primarily related to tumor biology and is associated with a significant decrease in overall survival (2). Treatment for recurrent RPS is individualized through multidisciplinary discussions (3). Research strategies are under development to expand genomic screening to guide treatment and improve the outcomes of patients with sarcoma (4, 5). Alterations in DNA damage repair (DDR) pathway genes have been associated with sarcoma development (6). Nacev et al. (7) characterized genomic alterations in a large cohort of 2,138 patients with 45 sarcoma subtypes by leveraging an institution-wide tumor genomic profiling initiative. They found that the frequency of DDR gene alterations was 10% in LMS. One of the altered genes across subtypes was *FANCA* (0.6%). Chudasama (8) observed deleterious aberrations in LMS were observed in multiple components of the homologous recombination repair (HRR) pathway, with high frequencies in *PTEN* (57%), *BRCA2* (53%), *ATM* (22%). Additionally, aberrations were also found in members of the Fanconi anemia complementation groups, specifically *FANCA* (27%) and *FANCD2* (10%).The most promising approaches for advanced leiomyosarcoma (LMS) include those targeting the DDR pathway (9). Research demonstrates FANCD2 plays a role in its resistance to treatment in alveolar rhabdomyosarcoma and a specific inhibition of FANCD2 leading to increased sensitivity to radiation in the PAX3-FOXO1 fusion gene cell line (10). However, there is still a lack of research on the effects of *FANCD2* deletion and genetic mutations on tumor sensitivity to radiotherapy. In this study, we present the clinical journey of a recurrent RLMS patient with a *FANCD2* gene mutation who underwent multiple radioactive iodine-125 (I-125) brachytherapy. This case highlights the potential of clinical molecular diagnostics in improving the management of radiation therapy for recurrent RLMS.

## Case report

**Figure 1:**
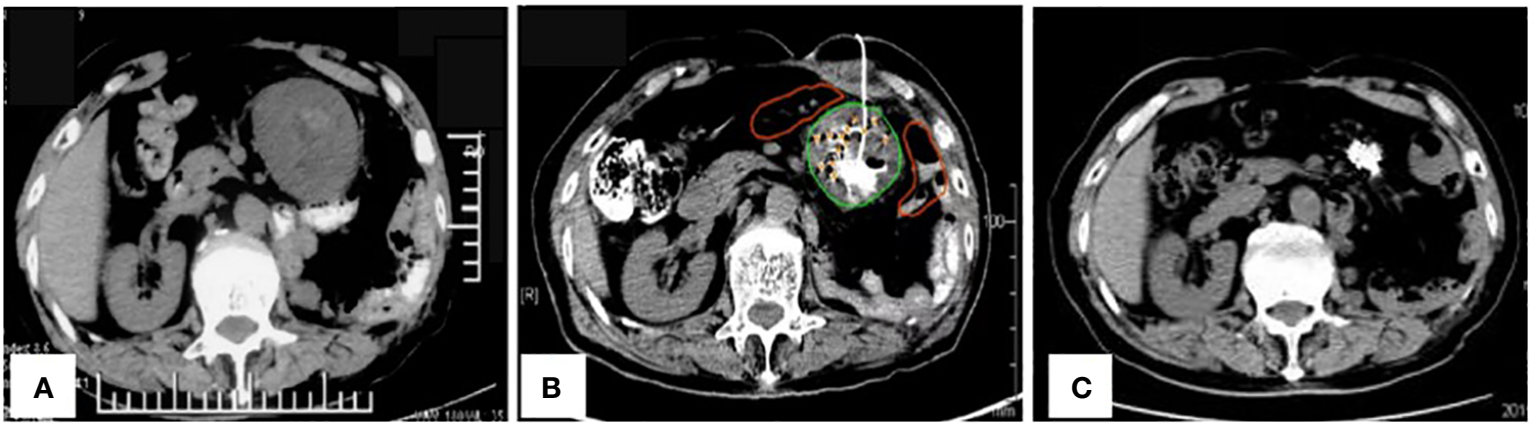
**(A)** Pre-operative imaging showed a recurrence in the left upper abdomen; **(B)** Placement of a drainage tube during the operation; **(C)** A complete response of the tumors in the left upper abdomen after six months and three iodine-125 seed implantation sessions.

**Figure 2:**
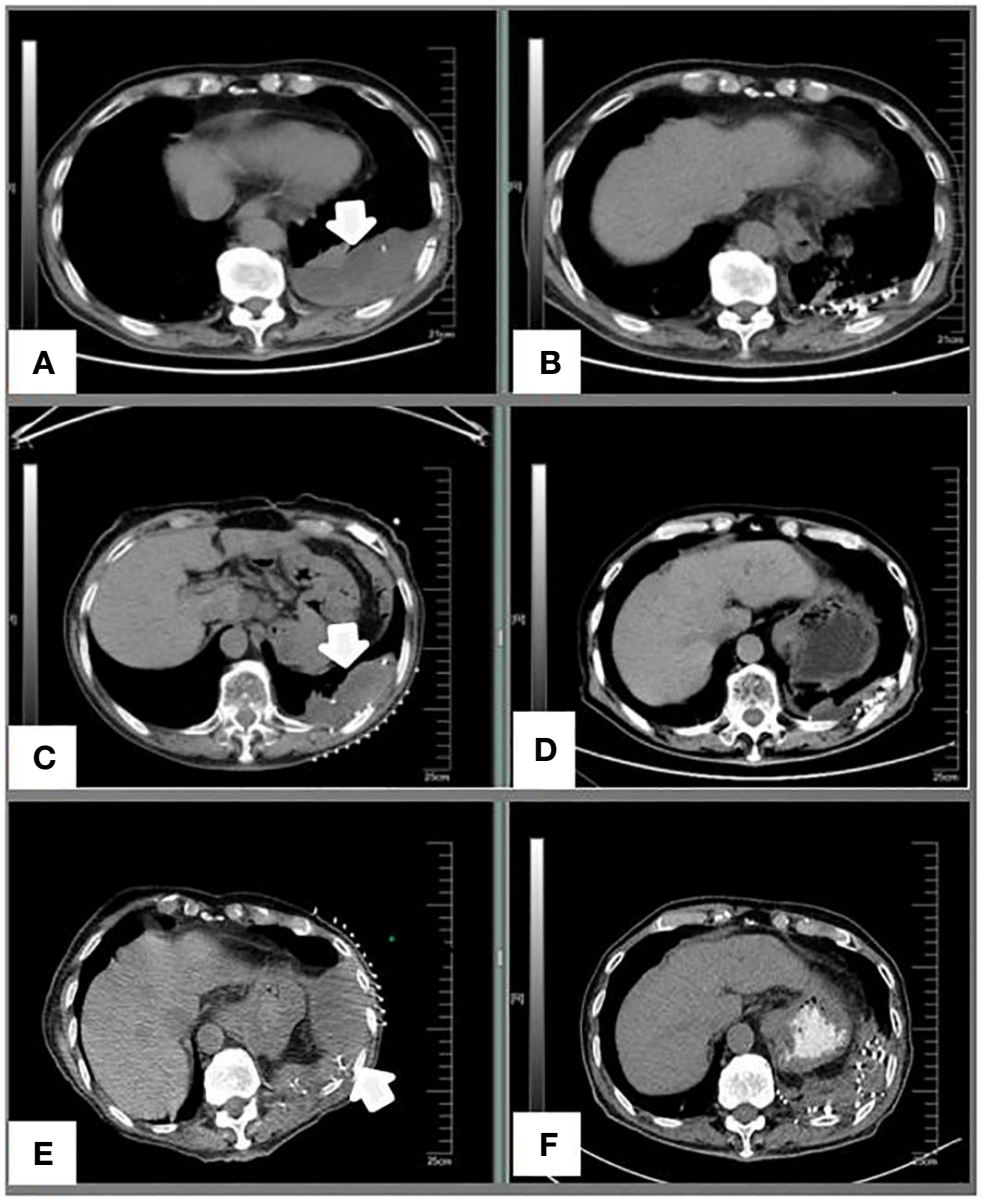
**(A)** A preoperative image of the left pleural metastases in September 2016. **(B)** Five months after I-125 seed implantation; **(C)** Progression of the left pleural metastases before treatment in January 2018. **(D)** Three months after I-125 seed implantation; **(E)** Re-progression of the left pleural metastases before treatment in November 2018. **(F)** Three months after I-125 seed implantation.

**Figure 3:**
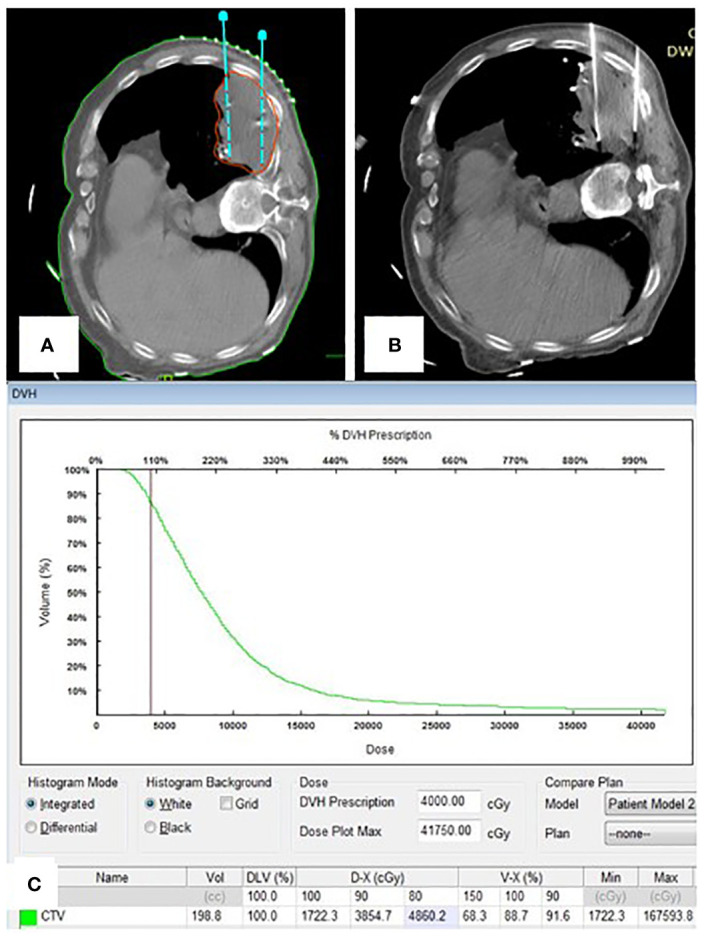
**(A)** Portion of pre-plan images; **(B)** Portion of real-time CT guided 125I seed implantation; **(C)** Dose-volume histogram of 125I seeds postoperative.

A 78-year-old male was admitted to our hospital in May 2015 with a history of three surgeries for retroperitoneal leiomyoma between 2001 and 2013. He had a recurrence 17 months after the last operation. A computed tomography (CT) scan revealed a tumor in the left upper abdomen measuring 12.9 × 6.9 × 6.6 cm. The patient experienced abdominal pain with a numeric rating scale (NRS) for pain score above 5. He patient refused further surgery and chemotherapy. To shrink tumors and relieve pain, I-125 radioactive seed implantation was performed under CT guidance, implanting 30 seeds once every 14 days. Ninety seeds (0.5 mCi) were implanted into the tumor bed, with a D90 [dose received by 90% of the gross tumor volume (GTV)] of 64.8 Gy. A drainage tube was provisionally installed, and 800 mL of reddish liquid was removed. A complete response of the left upper abdomen tumor was achieved after four months (**Figure 1**), and the NRS for pain score decreased to zero. However, a new metastatic lesion appeared in the left iliac fossa. The repeated local tumor recurrences were controlled by repeated implantations of 30–59 I-125 radioactive seeds with a D90 range of 15–65 Gy (**Figures 2**, **3**). The specific treatment details are shown in **Table 1**. The patient had no serious complications during or after the seed implantations and was discharged from hospital 1-2 days after the seed implantations every time.

**Table 1:** Lesion characteristics and treatment modality and efficacy.

Date	Tumor location	Seed number	GTV D90 (Gy)	Response
2015/5/19	Left upper abdomen	30	15	CR
2015/6/8		30	36	
2015/7/2		30	64.8	
2015/11/2	Left iliac fossa	26	32	PR
2016/4/15	L1 vertebra	30	26	PR
2016/9/27	Left pleural membrane	59	38	PR
2016/11/1	Progression of left iliac fossa tumor	30	20	PR
2016/12/6		30	42	
2017/7/24	L2 vertebra	30	21	PR
2017/8/29	Recurrent left upper abdomen tumor	26	54	CR
2017/9/25	Re-progression of left iliac fossa tumor	30	40	PR
2017/11/7	Progression of L1 vertebra tumor	30	46	PR
2018/1/19	Progression of left pleural membrane tumor	38	41	PR
2018/5/15	Nearby the right iliac vessels	30	45	PR
2018/8/1	Re-progression of left iliac fossa tumor 1	37	46	CR
2018/9/18	Re-progression of L1 vertebra tumor	30	38	PR
2018/11/5		30	50	
2018/11/21	Re-progression of left pleural membrane tumor	30	42	PR
2019/1/3	Re-progression of L1 vertebra tumor 1	40	57	PR
2019/2/18	Re-progression of left iliac fossa tumor 2	32	54	PR
2019/3/12	L2 vertebra and appendix	34	41	–

Complete response (CR) was defined as the complete disappearance of the target. Partial response (PR) was defined as at least a 30% decrease in the sum of diameters of target lesions.

In January 2018, a left pleural progression (**Figure 2**) biopsy was performed for genetic assessment during seed implantation. The pathology results indicated a low-grade malignant leiomyosarcoma, consistent with the diagnosis. Targeted DNA and RNA sequencing of a panel of 422 genes revealed an *FANCD2* frameshift mutation involving c.451_452delAA (p.K151fs) and c.3917delT (p.L1306). The patient refused systemic treatment. Subsequently, the patient’s mental condition deteriorated, refusing to re-enter the hospital for further medical treatments. The patient died from a self-administered drug overdose in September 2019, with an overall survival of 18 years. The patient underwent over 20 I-125 radioactive seed implantations without causing radiation-associated damage and lived for over four years after being deemed unsuitable for surgical resection.

## Discussion

The RPS recurrence intervals gradually shorten with the increase in the number of recurrences, and patients often die due to the local influence of recurrent tumors (11, 12). Therefore, reducing the local burden of recurrent RPS is critical to patient survival. Radiation treatment is generally considered beneficial for controlling local tumors. However, due to large volume of RPS tumors and their proximity to surrounding normal tissues, the risk of radiation-associated toxicity is substantial, reaching grade ≥3 toxicity in >30% of the cases (13, 14). Radioactive seed implantation represents a solution to mitigate healthy tissue damage. I-125 implantation is advantageous as brachytherapy inflicts less radiation damage to adjacent structures such as the bowel and genitourinary tract (15). Good local control, the goal of I-125 implantation for RPS, has been achieved (16). The I-125 seeds deliver continuous low-dose radiation, making them possibly more effective than daily pulsed high-dose irradiation in treating the hypoxic portion of large, slow-growing necrotic tumors (17). In the case of this patient with recurrent RLMS and such tumors, the repeated implantation of I-125 seeds for continuous radiotherapy proved beneficial. For certain tumors, repeated I-125 seed implantation could deliver better curative effects than external radiation therapy (18).

The literature indicates that the average dose for recurrent retroperitoneal sarcoma was reported to be 160 Gy (17). However, this research found that a D90 range of 15–65 Gy also yielded excellent results. The extraordinary response to low-dose radiation in this case might be attributed to the patient’s unique genetic background, the *FANCD2* gene mutation. The Fanconi anemia pathway has been shown to be triggered during DNA replication to repair DNA damage (19). FANCD2 is the focal center of the Fanconi anemia signaling system that repairs DNA damage and safeguards genomic stability (20). The *FANCD2* gene has 1,451 nucleotides and 44 exons, is located on chromosome 3p25.3, and has a mutation frequency of roughly 3% (21). Patients with Fanconi anemia are sensitive to ionizing radiation (22). Knocking down *FANCD2* gene expression increases cancer cell sensitivity to gamma rays (23). Some studies have shown that silencing *FANCD2* could greatly improve ionizing radiation sensitivity *in vitro* (24, 25). This study was the first to show the effect of *FANCD2* mutations on the clinical sensitivity of RLMS to radiotherapy. The effectiveness of low-dose radiotherapy in sarcoma has been previously reported in a case in which SS18-POU5F1 sarcoma had a quick, robust, but transient clinical response to low-dose radiation, while it showed no response to various systemic therapies, including immune checkpoint inhibitors, angiogenesis inhibitors, and chemotherapy (26). From sarcoma’s clinical molecular diagnostics standpoint, the good response to low-dose radiation is an intriguing finding. This would be a fruitful area for further work.

This case report had several limitations. First, systemic treatment was limited, especially in combination with immunotherapy. Second, the local CR helped prolong the recurrence interval. The difficulty in avoiding radiation-induced complications in the peritoneal cavity with an increasing number of seeds limited our ability to find an optimum dose. The multiple local progressions might be related to insufficient local doses. Third, the patient died from a self-administered drug overdose.

We reported repeated I-125 seed implantations applied in recurrent RLMS with excellent results that might be attributed to the patient’s unique genetic background, the *FANCD2* gene mutation. RLMS displays significant clinical and biologic heterogeneity. The future of RLMS treatment is contingent upon a greater understanding of tumor biology and the continued development of molecular markers. Future studies should investigate *FANCD2* gene mutations in RLMS, as they might render it vulnerable to radiation.

## Data availability statement

The original contributions presented in the study are included in the article/supplementary material. Further inquiries can be directed to the corresponding author.

## Ethics statement

The studies involving humans were approved by Medical Ethics Committee of Hebei general Hospital. The studies were conducted in accordance with the local legislation and institutional requirements. The human samples used in this study were acquired from Postoperative puncture specimens of patients. Written informed consent for participation was not required from the participants or the participants’ legal guardians/next of kin in accordance with the national legislation and institutional requirements. Written informed consent was obtained from the individual(s) for the publication of any potentially identifiable images or data included in this article.

## Author contributions

XL: Writing – original draft. JZ: Writing – review & editing, Data curation. XD: Writing – review & editing, Data curation. GC: Writing – review & editing, Supervision. HZ: Writing – review & editing. JW: Writing – review & editing.
